# Clinical significance of oxidative stress markers as angioinvasion and metastasis indicators in papillary thyroid cancer

**DOI:** 10.1038/s41598-023-40898-9

**Published:** 2023-08-22

**Authors:** Angelika Buczyńska, Iwona Sidorkiewicz, Maria Kościuszko, Agnieszka Adamska, Katarzyna Siewko, Janusz Dzięcioł, Piotr Szumowski, Janusz Myśliwiec, Małgorzata Szelachowska, Anna Popławska-Kita, Adam Krętowski

**Affiliations:** 1 Clinical Research Centre, Medical University of Bialystok, 15-276 Białystok, Poland; 2 Clinical Research Support Centre, Medical University of Bialystok, Ul. M. Skłodowskiej-Curie 24a, 15-276 Białystok, Poland; 3 Department of Endocrinology, Diabetology and Internal Medicine, Medical University of Bialystok, 15-276 Białystok, Poland; 4 Department of Human Anatomy, Medical University of Bialystok, 15-276 Białystok, Poland; 5 Nuclear Medicine, Medical University of Bialystok, 15-276 Białystok, Poland

**Keywords:** Thyroid cancer, Cancer screening

## Abstract

Angioinvasion remains the important prognostic feature in papillary thyroid cancer (PTC) patients. Literature data indicates several markers that may be associated with oxidative stress and/or angioinvasion. Therefore, we assessed the utility of selected parameters in angioinvasion and metastasis screening in serum of PTC patients. Serum antioxidant capacity (TAC) and sirtuin 3 (SIRT3) levels were decreased (all *p* < 0.05) and both DNA/RNA oxidative stress damage products (DNA/RNA OSDP) and malondialdehyde (MDA) levels were increased in PTC patients with angioinvasion and metastasis (study group) when compared with PTC patients without these features (all *p* < 0.01). The highest screening utility in differentiation between angioinvasion and metastasis presence and absence in PTC patients was presented for DNA/RNA OSDP (AUC = 0.71), SIRT3 (AUC = 0.70), and TAC (AUC = 0.67) (all *p* < 0.05). Our study suggests that peripheral concentration of oxidative stress markers could be useful as angioinvasion and metastasis indicator in PTC patients.

## Introduction

Papillary thyroid cancer (PTC) is one of the most commonly occurring endocrine neoplasms worldwide^1^. PTC originating from the follicular epithelium usually presents a good prognosis with an excellent survival rate. However, the diagnosis of aggressive types, manifested by local invasion, recurrence, and distant metastases, is still a significant clinical problem^2^. Angioinvasion has been identified as an important and independent prognostic factor in many cancers, including PTC. The diagnosis of thyroid cancer dedifferentiation with local angioinvasion is of great importance in further clinical management^3^. The molecular criteria for angioinvasion have varied both in practice as well as in studies assessing the clinical significance of these findings. This inconsistency may be attributed to application of inappropriate criteria^4^.

Oxidative stress is predicted to have a major impact both in the development and the progression of thyroid cancer, since, due to the process of hormonogenesis, the thyroid gland is exposed to a high level of reactive oxygen species (ROS). What should be emphasized, PTC is the most prevalent thyroid cancer associated with oxidative stress^5^. It has been demonstrated that ROS promote angiogenesis, either directly or via the generation of active oxidation products, including peroxidized lipids^6,7^.

Several markers may be associated with oxidative stress and/or angioinvasion. The total oxidative stress (TOS) and antioxidant capacity (TAC) are used to estimate the general oxidation status and antioxidant ability of the body, respectively. Malondialdehyde (MDA), the product of lipid peroxidation, is a commonly used as biological marker of this process. DNA/RNA oxidative stress damage products (DNA/RNA OSDP) show the extent of nucleic acid oxidative modifications^8,9^. Moreover, growing literature data links oxidative stress with DNA methylation in cancer development and progression. Nuclear factor kappa B (NF-κB) and interleukin-6 (IL-6) play a significant role in immune and inflammatory processes. In turn, sirtuin 1 and 3 (SIRT1 and SIRT3) and forkhead box protein 01 (FOXO) act as regulators of important metabolic pathways including redox homeostasis^10^. Protein 53 (p53), 5′-AMP-activated protein kinase catalytic subunit alpha-1 (PRKAA1), and peroxisome proliferator-activated receptor gamma coactivator 1-alpha (PGC1α) regulate metabolic reprogramming and antioxidant/mitochondrial function^11^. Their usefulness in angioinvasion screening in PTC remains to be elucidated.

It can be assumed the interplay between oxidative stress, chronic inflammation, and angiogenesis in cancer progression. Thus, our hypothesis is that circulating oxidative stress markers, inflammation and/or angiogenesis related parameters can be helpful in angioinvasion screening in order to support clinical evaluation in questionable PTC cases. Thus, the aim of this study was to assess the utility of selected parameters in angioinvasion and metastasis screening in serum of PTC patients.

## Results

### Biochemical characterization

To test the working hypothesis the group of PTC patients was divided into 2 subgroups: PTC patients with both angioinvasion and metastasis (study group) and PTC patients without these features (reference group, considered as very low-risk PTC group according to American Thyroid Association guidelines)^12^ **(**Table1**).**

**Table 1:** The characteristics of PTC patients.

	Study group
Number of patients	80
Median age (upper and lower quartiles)	54 (51.41; 64.22)
Sex	M: 22
	F: 58
Menopausal status	
Premenopausal	13
Postmenopausal	45
Stage (TNM)	pT1a:20
	pT1a(m):11
	pT1b: 15
	pT1b(m): 6
	pT2: 16
	pT3/pT4: 12
Patients diagnosed with angioinvasion and metastasis	56
Patients without angioinvasion and metastasis	24

F, female; M, male; (m), multifocal; p, pathological; TNM—Cancer tumor-node-metastasis classification (based on the characteristics of primary tumor site (pT)), pT1a- Tumor size ≤ 1 cm in greatest dimension limited to the thyroid, pT1b—Tumor > 1 cm but ≤ 2 cm in greatest dimension, limited to the thyroid, pT2- Tumor size > 2 cm but ≤ 4 cm, limited to the thyroid, pT3/pT4-Tumor size > 4 cm, with gross extrathyroidal extension.

The cholesterol (CHOL) and low-density lipoprotein (LDL) concentrations were increased, and high-density lipoprotein (HDL) was decreased in study group when compared with reference group (*p* < 0.05; *p* < 0.001; *p* < 0.05; respectively). Yet, other parameters did not differ among groups (Table 2).

**Table 2:** The biochemical profiles of the study and reference groups.

Parameter	Unit	Study group (n = 56)	Reference group (n = 24)	*P*-value
		Median (range)	Median (range)	
CHOL	mg/dL	231	194.5	**0.029**
		(162–363)	(101–282)	
LDL	mg/dL	160.5	117.4	** < 0.001**
		(88–314)	(55.7–192)	
TG	mg/dL	139.3	127.5	0.921
		(40–411)	(45–211)	
HDL	mg/dL	50.47	59.21	**0.043**
		(31–90)	(37–114)	
GLUCOSE	mg/dL	96.05	96.44	0.508
		(77–154)	(69–248)	
CRP	mg/L	2.15	3.4	0.587
		(0.4–8.8)	(0.2–32.6)	
25-OH VIT D	ng/ml	27.28	27.19	0.597
		(10.6–51.1)	(8.2–82.6)	
TGB	ng/ml	4.4	1.3	0.054
		(0.04–37.05)	(0.04–3.2)	
TGBAb	IU/mL	11.22	8.99	0.713
		(0.6–132.4)	(0–185)	
TSH	μIU/mL	0.76	4.38	0.092
		(0.05–3.83)	(0.09–7.3)	
FT3	pg/mL	2.64	2.64	0.883
		(1.36–3.76)	(1.0–6.27)	
FT4	ng/dL	1.2	1.18	0.941
		(0.54–2.14)	(0.4–1.82)	

Bold indicates that the values given are statistically significant.

CHOL, cholesterol; CRP, C-reactive protein; PTC, papillary thyroid cancer; fT3, free triiodothyronine; fT4, free thyroxine; HDL, high-density lipoprotein; LDL, low-density lipoprotein; TG, triglyceride; TGB, thyroglobulin; TGBAb, antithyroglobulin antibodies; TSH, thyroid-stimulating hormone; VIT D, vitamin D.

### Oxidative stress and PTC angioinvasion and metastasis

To evaluate the significance of oxidative stress in angioinvasion and metastasis features in PTC, several circulating oxidative stress-related markers were measured in PTC patients. Thus, patients with angioinvasion and metastasis were considered as the study group and patients without these characteristics constituted the reference group. Serum TAC and SIRT3 levels were decreased (all *p* < 0.05) and both DNA OSDP and MDA levels were increased in study group when compared with reference PTC patients (all *p* < 0.01). The study and reference groups did not differ in terms of TOC, SIRT1, DNA methylation, FOXO, PRKAA1, PGC1α, p53, NF-κB, and IL-6 (all *p* > 0.01) (Table 3).

**Table 3:** The oxidative stress markers measurement in PTC patients with angioinvasion and metastasis (study group) versus PTC patients without angioinvasion and metastasis (reference group).

Parameter	Unit	Study group (n = 56)	Reference group (n = 24)	*P*-value
		Median (range)	Median (range)	
TOC	umol/l	444	451	0.826
		(223–1009)	(123–882)	
TAC	umol/l	225	276	**0.036**
		(190–324)	(171–373)	
MDA	µM	174	97	**0.049**
		(31–650)	(3–373)	
DNA/RNA OSDP	pg/mL	611	510	**0.008**
		(405–750)	(310–684)	
IL-6	pg/mL	1.8	1.56	0.266
		(0.9–4.4)	(0.2–5.7)	
SIRT1	ng/mL	4.5	4.0	0.124
		(3.5–5.6)	(3.1–9.3)	
SIRT3	ng/mL	4.5	5.8	**0.011**
		(1.7–7.6)	(2.9–34.3)	
NF-κB	pg/mL	196	157	0.537
		(133–486)	(127–674)	
FOXO	pg/mL	41	59	0.547
		(27–289)	(26–413)	
DNA methylation	ng/ml	91	91	0.947
		(66–108)	(79–110)	
PRKAA1	pg/mL	86	78	0.179
		(35–200)	(14–247)	
PGC1α	pg/mL	57	78	0.849
		(15–513)	(14–248)	
p53	pg/mL	203	207	0.675
		(101–2619)	(110–3163)	

Bold indicates that the values given are statistically significant.

IL-6, interleukin 6; FOXO, Forkhead box O family member proteins; NF-κB, nuclear factor kappa B; MDA, malondialdehyde; p53, protein 53; SIRT1, Sirtuin 1; SIRT3, Sirtuin 3; TAC, total antioxidant capacity; TOC, total oxidative capacity; PRKAA1 5′-AMP-activated protein kinase catalytic subunit alpha-1; PGC1α, peroxisome proliferator-activated receptor gamma coactivator 1-alpha; MDA, malondialdehyde, DNA/RNA DNA/RNA oxidative stress damage products.

### Angioinvasion and metastasis indicators

**Figure 1:**
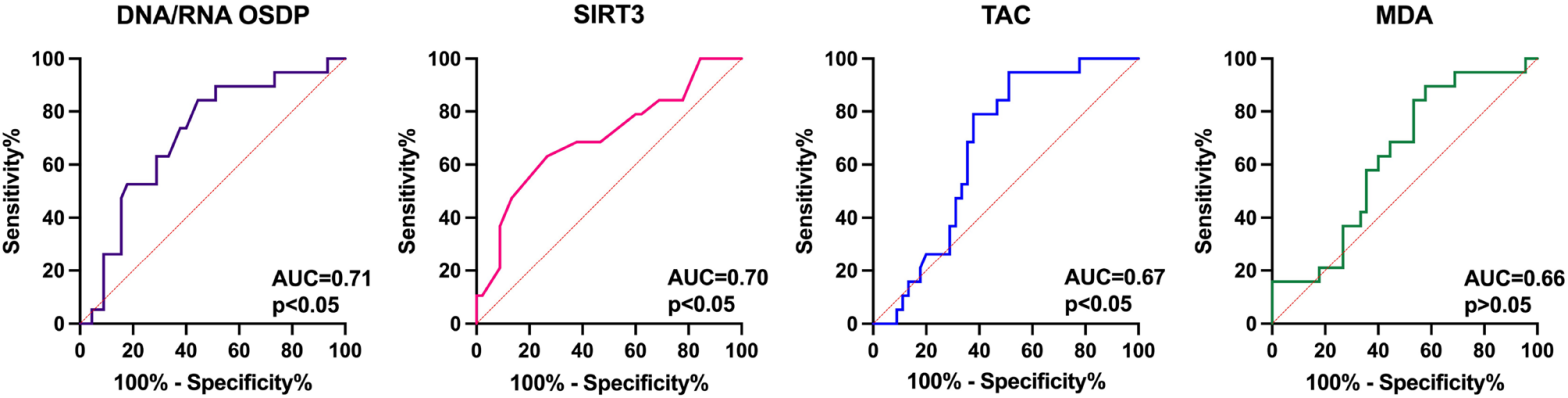
The screening utility of selected oxidative stress markers in angioinvasion and metastasis confirmation in PTC patients based on ROC analysis.

Furthermore, to assess the significance of TAC, SIRT3, DNA/RNA OSDP, and MDA as angioinvasion and metastasis indicators among PTC patients, the ROC curve analysis was performed. The highest clinical utility in angioinvasion and metastasis detection in PTC patients was observed for DNA/RNA OSDP (AUC = 0.71), SIRT3 (AUC = 0.70), TAC (AUC = 0.67) (all *p* < 0.05) measurements. However, the AUC for MDA (AUC = 0.66), somewhat high, did not reach significance when compared with AUC = 0.50 (*p* > 0.01) (Fig. 1).

### Correlation

**Figure 2:**
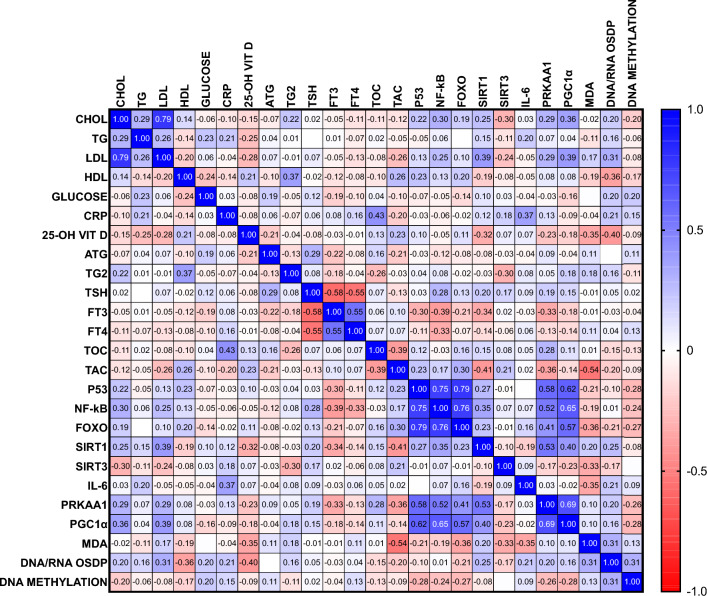
The studied parameters correlation; IL-6 interleukin 6, FOXO Forkhead box O family member proteins, NF-κB -nuclear factor kappa B, MDA malondialdehyde, P53 protein 53, SIRT1 Sirtuin 1, SIRT3 Sirtuin 3, TAC total antioxidant capacity, TOC total oxidative capacity, CHOL cholesterol, CRP C-reactive protein, PTC papillary thyroid cancer, fT3 free triiodothyronine, fT4 free thyroxine, HDL high-density lipoprotein, LDL low-density lipoprotein, TG triglyceride, TGB thyroglobulin, TGBAb antithyroglobulin antibodies, TSH thyroid-stimulating hormone, 25-OH VITD–25-OH vitamin D, PRKAA1 5'-AMP-activated protein kinase catalytic subunit alpha-1, PGC1α peroxisome proliferator-activated receptor gamma coactivator 1-alpha, MDA malondialdehyde, DNA/RNA DNA/RNA oxidative stress damage products.

A Spearman correlation analysis was performed to study the relationship between biochemical parameters in total group. The conducted correlation analysis included both groups: the study group, consisting of PTC patients with angioinvasion and metastasis, and the reference group, without the angioinvasion and metastasis, to assess the relationship between studied parameters in PTC patients (Fig. 2).

Considering all the biochemical parameters in PTC patients and their significant associations, strong positive correlation between p53 and NF-κB (r = 0.75; *p* < 0.001) and between p53 and FOXO (r = 0.79; p < 0.01) was shown. Moreover, medium negative correlation between TAC and MDA measurements was demonstrated (r = − 0.54; *p* < 0.001). Medium positive correlation between p53 and PRKAA1 (r = 0.58; *p* < 0.01) and between NF-κB and PGC1α (r = 0.65; *p* < 0.01) was also observed. Additionally, medium positive correlation between PRKAA1 and PGC1α (r = 0.69; *p* < 0.05) was shown.

### Logistic regression analysis

Furthermore, to distinguish whether the oxidative stress markers originate from the cancer itself or are a systemic phenomenon caused by the resultant peripheral processes, the logistic regression analysis was performed. We demonstrated the association between both angioinvasion and metastasis presence and CHOL, LDL, and HDL concentrations in PTC patients (*p* < 0.05). Moreover, we also observed that both angioinvasion and metastasis presence influence serum TAC, DNA/RNA OSDP, SIRT3, and NF-κB concentrations in PTC patients (*p* < 0.05) (Table 4).

**Table 4:** The angioinvasion and metastasis regression analysis.

Parameter	B	SE	*p*	OR (95% CI)
The biochemical profile				
CHOL	0.016	0.007	< 0.05	1.016 (1.004–1.032)
LDL	0.033	0.011	< 0.05	1.034 (1.015–1.058)
TG	0.002	0.003	NS	1.002 (0.995–1.008)
HDL	− 0.041	0.022	< 0.05	0.960 (0.915–0.998)
GLUCOSE	− 0.001	0.012	NS	0.999 (0.968–1.022)
CRP	− 0.082	0.095	NS	0.921 (0.706–1.054)
25-OH VIT D	0.001	0.022	NS	1.001 (0.955–1.043)
TGB	0.238	0.355	NS	1.269 (0.984–2.678)
TGBAb	0.003	0.009	NS	1.003 (0.981–1.021)
TSH	− 0.046	0.050	NS	0.955 (0.822–1.025)
FT3	− 0.008	0.373	NS	0.995 (0.451–2.064)
FT4	0.207	0.876	NS	1.230 (0.216–7.119)
The oxidative stress markers				
TOC	− 0.0001	0.001	NS	0.100 (0.997–1.002)
TAC	− 0.014	0.006	< 0.05	0.986 (0.974–0.997)
MDA	0.004	0.002	NS	1.004 (0.100–1.008)
DNA/RNA OSDP	0.005	0.003	< 0.05	1.005 (1.000–1.011)
IL-6	0.097	0.227	NS	1.102 (0.691–1.718)
SIRT1	0.142	0.264	NS	1.152 (0.666–1.971)
SIRT3	− 0.458	0.200	< 0.05	0.633 (0.408–0.896)
NF-κB	− 1.636	2.418	< 0.05	0.195 (0.001–14.280)
FOXO	− 0.379	0.443	NS	0.684 (0.225–1.440)
DNA methylation	− 0.018	0.039	NS	0.982 (0.907–1.060)
PRKAA1	0.351	0.349	NS	1.421 (0.713–3.044)
PGC1α	0.619	0.533	NS	1.857 (0.654–5.470)
p53	− 0.0001	0.001	NS	1.000 (0.999–1.001)

CHOL cholesterol, CRP C-reactive protein, PTC papillary thyroid cancer, fT3 free triiodothyronine, fT4 free thyroxine; HDL, high-density lipoprotein; LDL, low-density lipoprotein; TG, triglyceride; TGB, thyroglobulin; TGBAb antithyroglobulin antibodies; TSH, thyroid-stimulating hormone; VIT D, vitamin D; IL-6, interleukin 6; FOXO, Forkhead box O family member proteins; NF-κB, nuclear factor kappa B; MDA, malondialdehyde, p53, protein 53, SIRT1, Sirtuin 1; SIRT3, Sirtuin 3; TAC, total antioxidant capacity; TOC, total oxidative capacity; PRKAA1 5′-AMP-activated protein kinase catalytic subunit alpha-1; PGC1α, peroxisome proliferator-activated receptor gamma coactivator 1-alpha; MDA, malondialdehyde; DNA/RNA DNA/RNA oxidative stress damage products; B beta, SE, standard error; p, *p*-value; OR, odd ratio.

## Discussion

The literature data based results assessing the predictive value of angioinvasion in distant metastasis development, especially in well-differentiated thyroid cancer, are discrepant^13–15^. This may be due to the inconsistent criteria for angioinvasion detection in endocrine tumors, especially thyroid cancer^13^. Assessment of angioinvasion in primary tumors is currently based upon examination of sections stained with haematoxylin and eosin. Moreover, several vascular markers have been characterized and studied to detect cancer angioinvasion, e.g., a commonly used endothelial marker CD31, platelet-associated protein CD61, and von Willebrand factor (factor VIII-related antigen)^16–19^.

There is clear evidence for a higher level of oxidative stress, including a compromised antioxidative defense system in cancer^20^. Janion et al.^21^ showed significantly lower serum MDA and TAS levels and higher TOS levels in the colorectal cancer (CRC) group compared to the control group. Oxidative stress has been also suggested as significant PTC risk factor and the level of ROS generation correlated with cancer aggressiveness^22^. Therefore, potential modulation of oxidative status may influence the cancer progression. It has been hypothesized that oxidative stress markers in thyroid tumors may have diagnostic, prognostic, and therapeutic relevance^22^. To date, little studies have been performed to establish the tools for angioinvasion detection in cancer. Thus, new potential circulating biomarkers indicative of angioinvasion and metastasis are needed.

Accumulating evidence suggest that ROS function as signaling molecules to mediate various growth-related responses including angiogenesis^23^. It has been observed that oxidative stress stimulates the secretion of angiogenic modulators in cancer cells via hypoxia-dependent and -independent pathways^24^. In our study, the level of serum oxidative stress markers was evaluated to assess the potential screening utility in PTC-related angioinvasion with metastasis. PTC patients with both angioinvasion and metastasis presented decreased serum TAC and SIRT3 and increased DNA OSDP and MDA levels than PTC patients without these features. The results indicate that oxidative stress is involved in angioinvasion development among PTC patients and TAC, SIRT3, DNA/RNA OSDP, and MDA could be potentially considered as PTC angioinvasion screening tools. TAC measures the peroxyl-scavenging capacity of the extracellular antioxidant system and indirectly reflects the level of oxidative stress. A low serum TAC has been reported to have a strong association with various cancers^25^. Reduced TAC in studied PTC patients when compared with reference PTC patients could reflect the consumption of antioxidants in free radical reactions by the cancer invasion. This suggests that TAC may have a protective role against angioinvasion and metastasis.

Based on MDA, lipid peroxidation was significantly increased in PTC patients with angioinvasion and metastasis compared with respective reference group. These results also point to increased oxidative stress in those patients. Furthermore, Maurya et al.^26^ demonstrated that pre chemotherapy serum MDA level was significantly higher in patients with primary ocular carcinoma having larger tumor and having lymph node metastasis than those without lymph node metastasis suggestive of its potential significance in cancer progression.

SIRT3, the major deacetylase in mitochondria, plays a crucial role in regulating glucose metabolism, modulating ROS, and limiting the oxidative damage in cellular components^27^. The roles of SIRT3 vary in different types of cancers and they have been thoroughly discussed. In some types of cancer SIRT3 functions as a tumor promoter by lowering ROS levels to maintain cell proliferation, other studies describe SIRT3 as a tumor suppressor, as it can promote apoptosis in cancer cells under stress conditions^28^. SIRT3 plays a crucial role by mediating interactions between mitochondria and intracellular signaling^29^. It has been observed that increased ROS production in SIRT3 null cells contributes to increased HIF1α stabilization leading to the transcriptional activation of multiple metastasis-related pathways^30,31^. Our study suggests that SIRT3 plays a role as angiogenic factor in PTC, however, its involvement in PTC progression is yet to be elucidated.

Interestingly, oxidative damage to nucleic acid has been associated with the development of various diseases including cancer^32–35^. 8-Oxo-7,8-dihydro-2’-deoxyguanosine, 8-oxo-7,8-dihydroguanosine, and 8-hydroxy-2′-deoxyguanosine (8-OHdG) are generated by the oxidative damage to DNA. Increased serum 8-oxodG concentration compared to the control group has been observed in CRC patients^21^. Moreover, it has been demonstrated increased levels of urinary 8-OHdG in CRC patients and patients with tumor metastasis, compared to healthy controls and patients without tumor metastasis, respectively^36^. This line of evidence suggest that peripheral concentration of DNA/RNA OSDP may be useful in noninvasive screening of angioinvasion and metastasis in cancer patients.

Oxidative stress can also activate a variety of transcription factors including NF-κB, FOXO, and p53. P53 is the most extensively studied tumor suppressor deregulated in most human cancers. In turn, NF-κB transcription factors play essential regulatory function in inflammation, cell proliferation, and apoptosis^37^. FOXO proteins are growth factor and stress regulated transcription factors known to cooperate with p53^38^. The reciprocal regulation of these transcription factors can influence several important cancer pathways: tumor cell metabolism, DNA damage, mitochondrial function, and ATP production^39^. Depending on the cellular context, the activation of NF-κB can have different related output to the p53 function. Our study confirmed strong positive correlation between p53 and NF-κB (r = 0.75; *p* < 0.001) and between p53 and FOXO (r = 0.79; *p* < 0.01) in PTC patients. FOXO mediates cellular oxidative stress to maintain metabolic stability by promoting transcription of several genes coding for antioxidant proteins^40^.

Moreover, medium negative correlation between TAC and MDA measurements was demonstrated (r = − 0.54; *p* < 0.001). As MDA is a marker for lipid peroxidation, the results indicate the imbalance between oxidant and antioxidant capacity in PTC, and, therefore, oxidative damage in cells and tissues. Positive correlation between p53 and PRKAA1 (r = 0.58; *p* < 0.01) and between NF-κB and PGC1α (r = 0.65; p < 0.01) was also observed in all PTC patients. Additionally, medium positive correlation between PRKAA1 and PGC1α (r = 0.69; *p* < 0.05) was shown. PRKAA1 is a major cellular energy sensor implicated in angiogenesis in hypoxia-induced diseases^41^. In turn, PGC1α plays a pivotal role in mitochondrial biogenesis and multiple other pathways (inflammation, endothelial dysfunction, and oxidative stress)^42^. As no difference in serum PRKAA1 and PGC1α concentration was observed between groups, it can be concluded that their peripheral level is not influenced by angioinvasion development as they are produced and exert effects locally.

Bauerle et al. demonstrated the key role of NF-κB in angiogenesis and growth of primary and metastatic thyroid cancer^43^. Since, considering these markers, no difference was observed between PTC patients with both angioinvasion and metastasis and PTC patients without these features, the associations need further study to evaluate their role in PTC invasion^44^. Targeting oxidative stress-sensitive pathways and transcription factors offers great promise for cancer prevention and therapy^45^. Nevertheless, the direct roles of ROS and distinct oxidative stress markers in angiogenesis in PTC patients remain to be elucidated. However, serum oxidative status marker levels are good indicators for the systemic oxidant/antioxidant status.

To assess the potential screening utility of the parameters, ROC curves were constructed. DNA/RNA OSDP (AUC = 0.71), SIRT3 (AUC = 0.70), and TAC (AUC = 0.67) have been demonstrated to differentiate between PTC patients with both angioinvasion and metastasis and PTC patients without these features (all *p* < 0.05). In our regression analysis results, a significant influence of angioinvasion and metastasis on various peripheral parameters, which showed statistical significance (*p* < 0.05), was observed. Serum parameters such as CHOL, LDL, HDL, TAC, OSDP, SIRT3, and NFκB showed significant association with the presence of both angioinvasion and metastasis in PTC patients. Thus, it can be concluded that the concentration change of these parameters originates from the cancer itselft. In previously conducted studies, we also observed a positive correlation between the concentration of MDA measured among advanced PTC patients and their lipid profile. This finding suggests that individuals with advanced PTC may be at risk of having deregulated lipid levels, which, in turn, is associated with the metastasis process^46,47^. On the other hand, in our analysis, it has been shown the difference in the MDA concentration between groups, however, our regression analysis did not demonstrate the association between angioinvasion and metastasis status and serum MDA level. Therefore, it can be assumed that the change in MDA concentration results from the peripheral oxidative stress rather than the cancer. It can be hypothesized that additional mechanisms involving lipids and MDA are associated with PTC progression, which need further detailed evaluation. Additional investigations on these peripheral metabolic processes will enable to better understand their role and potential clinical significance. Larger studies are also needed to confirm the results and assess the possibility to support of serum oxidative stress markers in challenging PTC screening.

To our knowledge, this is the first study assessing circulating oxidative stress markers as angioinvasion and metastasis indicators in PTC patients. Considering that the parameters were measured in serum, their peripheral concentration results not only from thyroid cancer tissue but also from the imbalance of homeostatic processes. Although the involvement of oxidative stress in PTC angioinvasion and metastasis seems to be important, our hypotheses need to be verified in further molecular studies.

Investigation of thyroid cancer progression from the overview of oxidative stress may be helpful in the understanding of the cancer etiology and may contribute to the development of new approaches to cancer therapy.

## Materials and methods

### Study design

This research was conducted at the Department of Endocrinology, Diabetology, and Internal Diseases, Medical University of Bialystok, Poland. All patients were diagnosed as having PTC based on clinical laboratory tests and ultrasound imaging, then confirmed by fine needle aspiration biopsy (FNAB), followed by histopathological examination. For this study 80 patients diagnosed with various stages of PTC after total thyroidectomy were enrolled (Table 1). To assess the clinical utility of the selected parameters as indicators of angioinvasion and metastasis, the study group comprised patients with PTC presenting angioinvasion and metastasis (study group), while the reference group consisted of PTC patients without these specific features (reference group). The angioinvasion was identified through post-thyroidectomy histopathological evaluation. To conduct this study, patient recruitment has been ongoing since 2018.

### Material and methods

Venous blood (5.5 mL) was obtained and centrifuged, with serum subsequent separation and then frozen at − 80 °C. The procedures were approved by the Local Ethics Committee of the Medical University of Bialystok, Poland, and written informed consent was obtained from each participant (R-I-002/491/2019).

The triglyceride (TG), LDL, HDL, CHOL, and C-reactive protein (CRP) concentrations were assayed using the enzymatic colorimetric method on a Roche C111 device (Roche Diagnostics, Basel, Switzerland). The thyroid-stimulating hormone (TSH), free triiodothyronine (fT3), free thyroxine (fT4), and antithyroglobulin antibody (TGBAb) concentrations were measured by a Roche E411 device (Roche Diagnostics, Sussex, UK) using the electrochemiluminescence (ECLIA) method. The TOS status was assessed using photometric immunodiagnostic assay (PerOx (TOS/TOC) kit, KC5100, 64625 Bensheim, Germany) and the TAC status was determined by photometric assay (ImAnOx (TAS/TAC) Kit, KC5200, 64625 Bensheim, Germany). P53, NF-κB, FOXO, SIRT1, PRKAA1, and PGC1α concentrations were determined using an enzyme-linked immunosorbent assay (ELISA) (Enzyme-linked Immunosorbent Assay Kit; Cloud-Clone Corp., Wuhan, China; SEA928Hu; SEB824Hu; SEA764Hu; SEE912Hu; SEA679Hu; SEH337Hu; respectively) according to the manufacturer’s instructions. SIRT3 and IL-6 concentrations were determined using ELISA method (Human Sirtuin 3, Thermofisher, CA 92008, Carlsband, USA; and Human IL-6; R&D Systems, D6050, Inc., Minneapolis, MN 55413, Canada. MDA was evaluated accordingly the ELISA method (Malondialdehyde ELISA kit, E-EL-0060, Elabscience, Wuhan, China). Moreover, DNA/RNA OSDP and DNA methylation were assessed using an immunoassay kit (DNA/RNA Oxidative Damage (High Sensitivity) ELISA Kit, Cayman Chemicals, 589320, Ann Arbor, Michigan, MI, USA; DNA Methylation ELISA Kit, Cayman Chemicals, 589324, Ann Arbor, Michigan, MI, USA).

Samples and controls were measured in duplicate using the blind analysis method in the same run.

### Statistical analysis

Statistical analyses were performed using GraphPad Prism v. 9.0 (GraphPad Software, Inc., San Diego, CA, USA). The lack of normality in data distribution using the Shapiro–Wilk test was demonstrated. Thus, the groups were compared using the nonparametric tests were used and *p* < 0.05 was considered statistically significant. To assess the screening utility, the receiver operating characteristic (ROC) curves were determined and the area under the ROC curves (AUC) was analyzed. Moreover, Spearman correlations and logistic regression analyses were performed.

### Ethical approval

The study was conducted in accordance with the Declaration of Helsinki and approved by the Ethics Committee of the Medical University of Bialystok, Poland (R-I-002/491/2019).

### Informed consent

Written informed consent has been obtained from the patients to publish this paper.

## Conclusions

Reporting angioinvasion as prognostic factor in PTC patients should be performed to ensure proper management and, therefore, screening tools to for objective evaluation of the process are of great importance. Our study confirmed the screening utility of serum oxidative stress markers as angioinvasion and metastasis indicators in PTC patients. The mechanisms that may connect to the angioinvasion process and oxidative stress in PTC need to be studied in depth.

## Data Availability

The datasets analyzed during the current study are available from the corresponding author on reasonable request.
